# Two ST11 *Klebsiella pneumoniae* strains exacerbate colorectal tumorigenesis in a colitis-associated mouse model

**DOI:** 10.1080/19490976.2021.1980348

**Published:** 2021-10-04

**Authors:** Ming-Ko Chiang, Pei-Yi Hsiao, Yen-Yi Liu, Hui-Ling Tang, Chien-Shun Chiou, Min-Chi Lu, Yi-Chyi Lai

**Affiliations:** a Department of Biomedical Sciences, National Chung Cheng University, Chia-Yi, Taiwan; b Department of Microbiology and Immunology, School of Medicine, Chung Shan Medical University, Taichung, Taiwan; c Department of Public Health, China Medical University, Taichung, Taiwan; d Department of Microbiology and Immunology, School of Medicine, China Medical University, Taichung, Taiwan; e Center for Research, Diagnostics, and Vaccine Development, Centers for Disease Control, Ministry of Health and Welfare, Taichung, Taiwan; f Division of Infectious Diseases, Department of Internal Medicine, China Medical University Hospital, Taichung, Taiwan; g Department of Internal Medicine, Chung Shan Medical University Hospital, Taichung, Taiwan

**Keywords:** *Klebsiella pneumoniae*, ST11, colorectal tumorigenesis, IL-10, M2 macrophages

## Abstract

Sequence type (ST) 11 is one of the major lineages of carbapenem-resistant *Klebsiella pneumoniae* (CRKP). Although the gastrointestinal (GI) carriage of CRKP predisposes individuals to subsequent infections, little is known for its impact on gut homeostasis. In this study, we investigated the association between ST11 CRKP colonization and colorectal cancer (CRC). Two ST11 CRKP, KPC160111 (KL47) and KPC160132 (KL64), were selected as the representative strains. We used azoxymethane (AOM) and dextran sodium sulfate (DSS) to initiate a colitis-associated CRC model. Both strains established prolonged colonization in the GI tract of the AOM-DSS-treated BALB/c mice and aggravated gut dysbiosis. Under this AOM-DSS-induced setting, ST11 *K. pneumoniae* colonization significantly promoted the growth and progression of colorectal adenomas to high-grade dysplasia. Numerous crypts were formed inside the enlarged adenomas, in which CD163^+^ tumor-associated macrophages accumulated. Similarly, ST11 *K. pneumoniae* also increased the population size of the CD163^+^ macrophages with the M2 phenotype in the peritoneal cavity of LPS-primed BALB/c mice. When applied to RAW264.7 cells, ST11 *K. pneumoniae* polarized the macrophages toward an M2 phenotype through the inhibition of IKK-NFκB and the activation of STAT6-KLF4-IL-10. Through the M2-skewing ability, ST11 *K. pneumoniae* promoted the accumulation of CD163^+^ macrophages in the adenomatous crypts to create an immunosuppressive niche, which not only accommodated the extended stay for its own sake but also deteriorated colorectal tumorigenesis.

## Introduction

*Klebsiella pneumoniae* is a concerning pathogen worldwide. As one of the ESKAPE, *K. pneumoniae* causes a wide range of infections associated with antimicrobial resistance, challenging to treat with limited therapeutic options.^1^ Over the past decades, drug-resistant *K. pneumoniae* has evolved from an extended-spectrum-β-lactamase (ESBL) producer into a carbapenem-resistant superbug. Carbapenems are the “last-line” treatment for infections caused by ESBL *K. pneumoniae*. The emergence and global spread of carbapenem-resistant *K. pneumoniae* (CRKP) poses a real threat to public health. Sequence type (ST) 11 constitutes the major CRKP in South America and Asia.^2–6^ Some lineages of ST11 have transformed into hypervirulent CRKP through acquiring a variant of virulence plasmids and had caused fatal outbreaks in China.^7,8^

Colorectal cancer (CRC) ranks third in mortality among all cancer, causing up to 690,000 annual deaths worldwide.^9^ Only 25% of CRC cases have a family history; the remaining 75% of affected individuals are not hereditary and occur spontaneously. *K. pneumoniae* is an etiological pathogen of pyogenic liver abscess (PLA). The association between CRC and *K. pneumoniae* PLA has been demonstrated.^10–12^ As revealed in an 11-year follow-up study in Taiwan, patients with PLA caused by *K. pneumoniae* had a significantly greater rate (adjusted hazard ration = 2.68) of subsequent CRC than those with non-*K. pneumoniae* PLA.^10^ *K. pneumoniae* can asymptomatically reside in the gastrointestinal (GI) tract and subsequently spreads to other organs, causing extra-intestinal infections.^13^ Although *Klebsiella* spp. are neither prime colonizers of the gut nor oral microbiomes in healthy individuals, the size of their population increases under certain circumstances, including the usage of antibiotics,^14^ inflammatory bowel diseases,^15^ and periodontitis.^16^ Enriched abundances of *Klebsiella* spp. in the GI tract positively correlated with several chronic disorders, including nonalcoholic fatty liver disease,^17^ atherosclerotic cardiovascular disease,^18^ and CRC.^19^ Although a recent study has demonstrated that an infant isolate *K. pneumoniae* 51–5 (ST1243) increases colon tumors in *Apc^Min^*^/+^; *Il10*^−/-^ mice,^20^ it is still unclear whether *K. pneumoniae* is considered a pro-carcinogen of CRC.

The GI carriage of CRKP can persist silently for years.^21^ During colonization, CRKP not only evolves resistance to novel antibiotics but may also interfere with gut homeostasis to predispose its carriers to tumorigenesis. Given that ST11 is a high-risk clone of CRKP in Asia, we examined the link between ST11 *K. pneumoniae* and CRC in an azoxymethane (AOM) and dextran sodium sulfate (DSS) mouse model. AOM-DSS has been widely used for the induction of CRC in mice.^22^ After absorption by colon epithelium, AOM chemically introduces O^6^ methylguanine adducts and causes G→A transition to initiate colorectal tumorigenesis.^23^ DSS is a sulfated polysaccharide with toxicity to colon epithelium that induces colitis mimicking features of inflammatory bowel disease (IBD).^24^ The utilization of AOM to mutate DNA followed by several cycles of DSS eliciting inflammation makes AOM-DSS an ideal initiation-promotion model of colitis-associated CRC. In this model, we demonstrated that two representative strains of ST11 *K. pneumoniae* established prolonged GI colonization, aggravated gut dysbiosis, promoted intratumoral accumulation of tumor-associated macrophages, and consequently exacerbated colorectal tumorigenesis.

## Results

### Two representative ST11 strains, KPC160111 and KPC160132, established GI colonization and exacerbated colorectal tumorigenesis in an AOM-DSS mouse model

ST11 *K. pneumoniae* could be phylogenetically divided into sub-lineages based on their capsular (K) loci. We downloaded 857 ST11 genomes (publicly available in GenBank in October 2019) and identified their K-locus (KL) types with Kaptive.^25^ KL64, KL47, and KL105 constituted the major of ST11, by 35%, 21%, and 12%, respectively (Supplementary Figure S1a). Over 90% of ST11_KL64 and ST11_KL47 *K. pneumoniae* carried at least one carbapenemase-coding gene, while less than 30% of ST11_KL105 were carbapenemase-positive (Supplemental Figure S1b). Because KL64 and KL47 were the dominant ST11 sub-lineages, we selected KPC160111 (ST11_KL47) and KPC160132 (ST11_KL64), which had been completely sequenced in our previous studies,^26,27^ as the representative strains of ST11 *K. pneumoniae* for the subsequent experiments.

**Figure 1.**
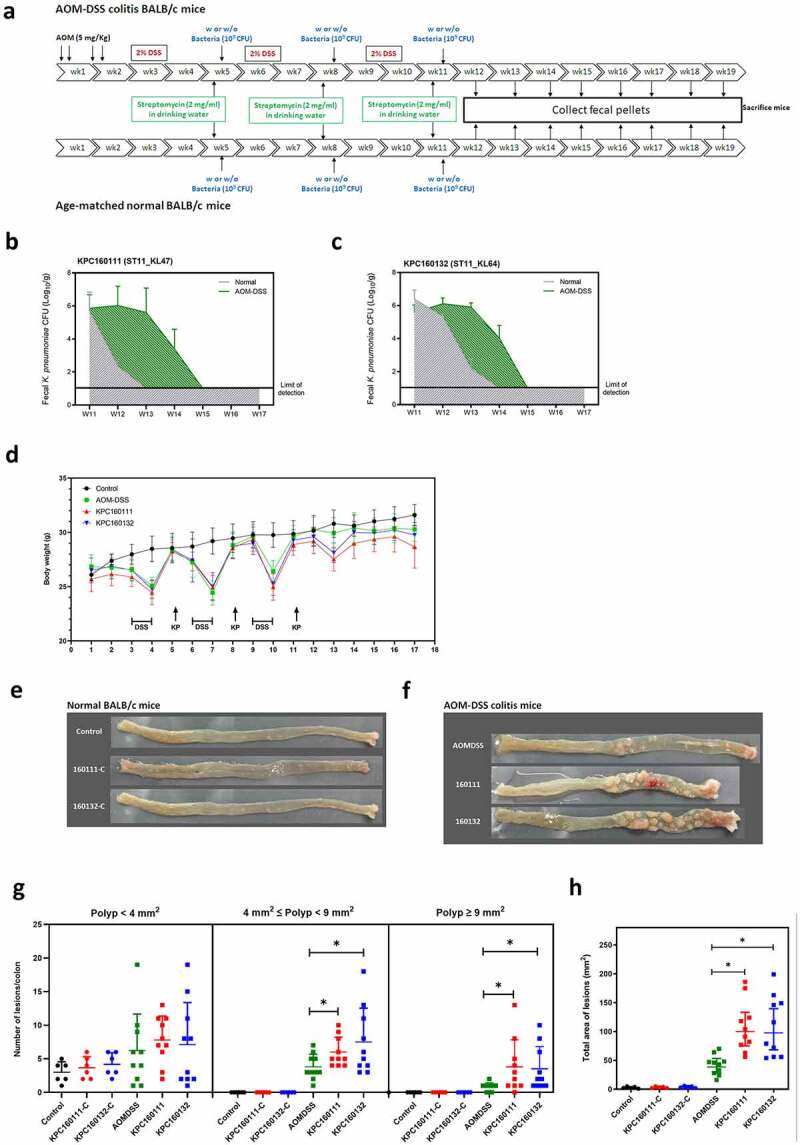
**ST11 *K. pneumoniae*, KPC160111 (ST11_KL47) and KPC160132 (ST11_KL64) exacerbated colorectal tumorigenesis in an AOM-DSS mouse model**. (a) The schematic timeline for the colitis model induced by azoxymethane (AOM) and dextran sodium sulfate (DSS) with and without KPC160111 or KPC160132 co-administration. Carbapenem-resistant *K. pneumoniae* CFUs in fecal pellets collected from individual mice were determined by counts on imipenem (10 μg/ml)-M9 minimal medium plates. Fecal loads of KPC160111 (b) and KPC160132 (c) in the AOM-DSS colitis mice (green area) are shown in comparison with that in the age-matched normal groups (a gray area). (d) Bodyweight of individual mice from the normal control (black circles), AOM-DSS-alone (green circles), AOM-DSS with KPC160111 (red circles), or KPC160132 (blue circles), was weekly recorded during the experiment period. Representative macroscopic appearance of the colonic lesions developed in the age-matched normal BALB/c mice (e) and in the AOM-DSS-treated mice (f) at week 17. Numbers of the polys (g), categorized into three classes, sporadic polyps (<4 mm^2^), small neoplastic polyps (4 ~ 9 mm^2^), and large adenomas (≥9 mm^2^), and the total area of colon polyps (h) were calculated for individual mice of each group. *P* values were determined by two‐tailed Student’s *t*-test between AOM‐DSS alone and AOM-DSS-KPC160111 or AOM-DSS-KPC160132. * P < .05

To investigate the role of KPC160111 (ST11_KL47) and KPC160132 (ST11_KL64) in the development of CRC, we established an AOM-DSS mouse model. The schematic timeline is shown in Figure 1a. Groups of normal or AOM-DSS-treated BALB/c mice were orally challenged with 1 × 10^9^ CFU of KPC160111 or KPC160132 at weeks 5, 8, and 11. Fecal CFUs of ST11 *K. pneumoniae* were determined once a week after the 3^rd^ bacterial inoculation at week 11. Compared to the age-matched normal groups, KPC160111 and KPC160132 maintained higher bacterial loads at the level of 10^2^–10^5^ CFU/g of feces for at least four weeks in the AOM-DSS-treated groups (Figure 1b, c). This result suggested that the GI-carriage of ST11 *K. pneumoniae* was significantly potentiated by the AOM-DSS treatment. Given that ST11 *K. pneumoniae* was nonpathogenic in the GI tract, as expected, the severity of colitis-associated symptoms (loss of body weight, diarrhea, and rectal bleeding) was not significantly exacerbated by KPC160111 or KPC160132 colonization (Figure 1d). All the mice survived the experimental period of 17 weeks. In the end, we sacrificed all the mice for colonic examination. In the absence of AOM-DSS, regardless of KPC160111 or KPC160132 colonization, all the mice of age-matched normal groups produced sporadic polyps in small size (<4 mm^2^), which were non-neoplastic (Figure 1e). In the AOM-DSS-treated groups, neoplastic polyps (≥4 mm^2^) were formed and primarily present in the middle to the distal colon (Figure 1f). Although all the AOM-DSS-treated mice developed neoplastic polyps, coadministration with KPC160111 or KPC160132 significantly increased the incidence and multiplicity of large neoplastic polys (≥9 mm^2^) (Figure 1g) and the total area of polyps (Figure 1h).

**Figure 2.**
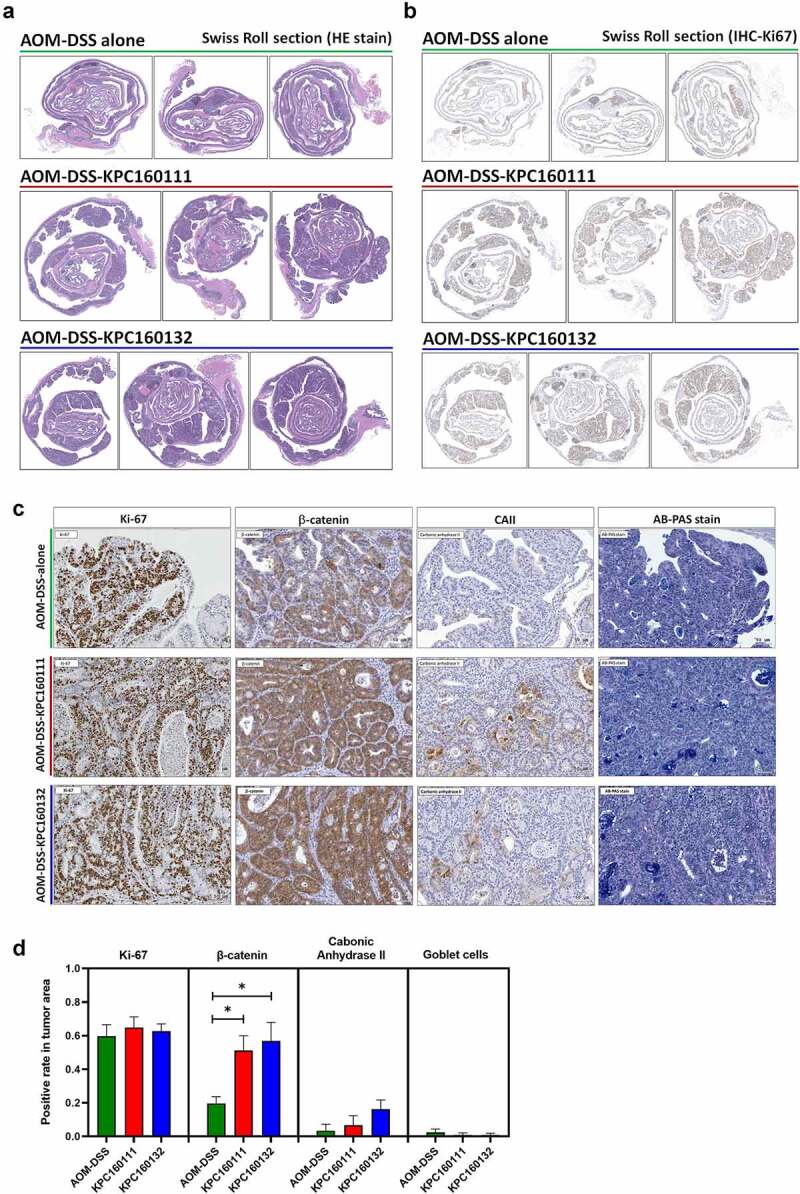
**Large tubular adenomas developed in the AOM-DSS mice co-administrated with ST11 *K. pneumoniae***. Consecutive sections of Swiss colon rolls from individual mice were stained with Hematoxylin and Eosin (H&E), Alcian Blue (AB) Periodic Acid Schiff (PAS) for the detection of goblet cells, and were immunostained with Ki-67, β-catenin, and carbonic anhydrase (CA) II. Representative images of whole-mount sections analyzed by H&E stain (a) and Ki-67 IHC (b) are presented. (c) Representative images, analyzed by Ki-67, β-catenin, CAII IHC and AB-PAS stain, of the large adenomas developed in the AOM-DSS mice with and without KPC160111 or KPC160132 co-administration. (d) Positive rates of cells with positive signals against Ki67, β-catenin, and CA II and goblet cells were quantified by HistoQuest Software at a high-powered field in 5 randomly selected areas in each of the tumors or corresponding control regions. Data are presented as mean ± SD (n = 30 in each group). *P* values were determined by two‐tailed Student’s *t*-test between AOM‐DSS alone and AOM-DSS-KPC160111 or AOM-DSS-KPC160132. * P < .05

**Figure 3.**
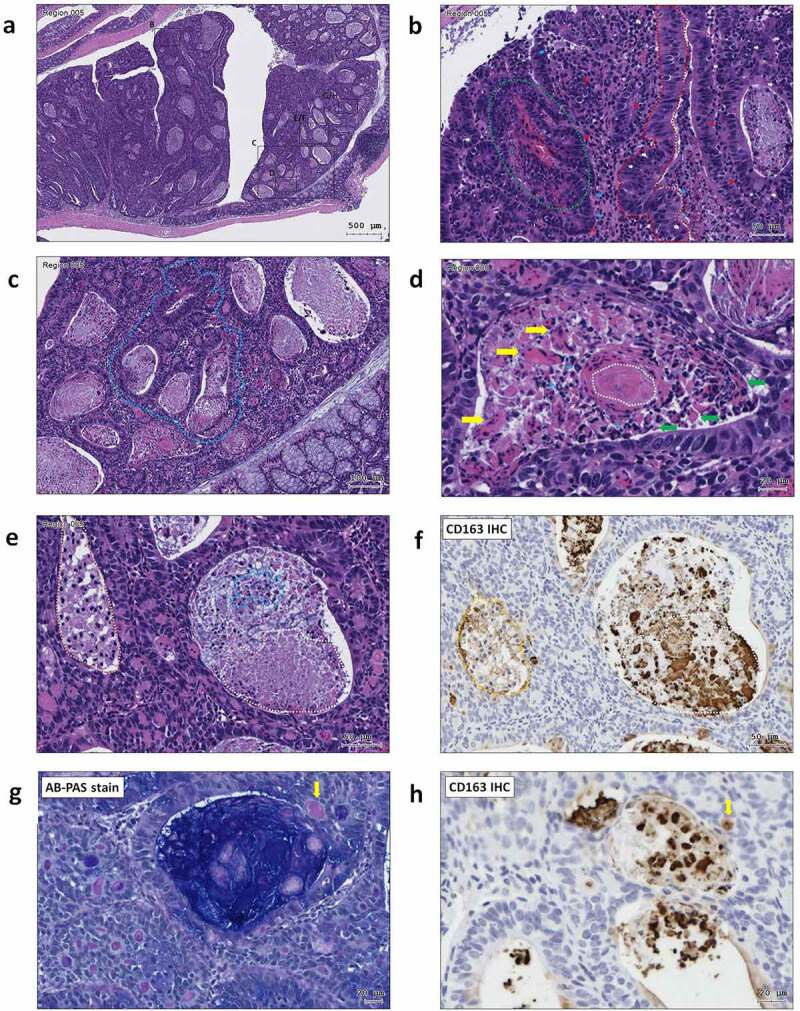
**ST11 *K. pneumoniae* accelerated adenoma progression to high-grade dysplasia in AOM-DSS mice**. (a) Representative images showing adenomas with high-grade dysplasia. The regions with histopathological features are framed and enlarged in B-H. (b) An adenoma region with full-thickness pseudostratification (red dash line), hemorrhage (green dash line), abnormal cells with enlarged and irregular nuclei (white arrow), infiltration of lymphocytes (red arrow) and neutrophils (blue arrow); (c) Cribriform (blue dash line) growth pattern of an adenoma; (d) A granuloma-like structure containing epithelioid macrophages (green arrow), giant cells (yellow arrow), and caseous necrosis (white dash line) in an adenomatous crypt; (e) Accumulation of foam cells (orange dash line), neutrophils (blue dash line), and macrophages (white dash line) in adenomatous crypts. CD163 immunostaining is shown in (f). Some of the macrophages with the red PAS positivity (g) were CD163^+^ (h) (yellow arrow)

For histopathological examination of polyps, sections of the entire colon Swiss roll were stained with hematoxylin and eosin (H&E) (Figure 2a). Despite the AOM-DSS-KPC160111 or KPC160132 mice produced much more large polyps (≥9 mm^2^), all the neoplastic polyps (≥4 mm^2^) which developed throughout the AOM-DSS groups belonged to tubular adenomas. Among the colorectal tumorigenic features analyzed, including the rates of cells positive for Ki-67 or β-catenin, suppression of carbonic anhydrase II, and the loss of goblet cells (Figure 2b,c), a significantly higher level of nucleo-cytoplasmic distribution of β-catenin was detected in the adenomas produced in the AOM-DSS-ST11 *K. pneumoniae* mice than that in the AOM-DSS-alone group (Figure 2d). Moreover, the ST11 *K. pneumoniae*-associated large adenomas (≥9 mm^2^) (Figure 3a) had features showing high-grade dysplasia, including full-thickness pseudostratification (Figure 3b; red dash line), hemorrhage (Figure 3b; green dash line), abnormal cells with enlarged and irregular nuclei (Figure 3b; white arrow), and cribriform (Figure 3c; blue dash line). Besides the cytological and architectural abnormalities, numerous crypts were formed inside the large adenomas which were exclusively presented in the AOM-DSS-ST11 *K. pneumoniae* groups (Figure 3a). The lumens of these adenomatous crypts were not empty but massively filled with macrophages, which were morphologically transformed to epithelioid cells, foam cells, or giant cells (Figure 3d,e). Caseous necrosis was found in the center of cell aggregates (Figure 3d; white dash line).

### *ST11* K. pneumoniae *promoted the accumulation of CD163^+^ IL-10^+^ macrophages in adenomatous crypts*

**Figure 4.**
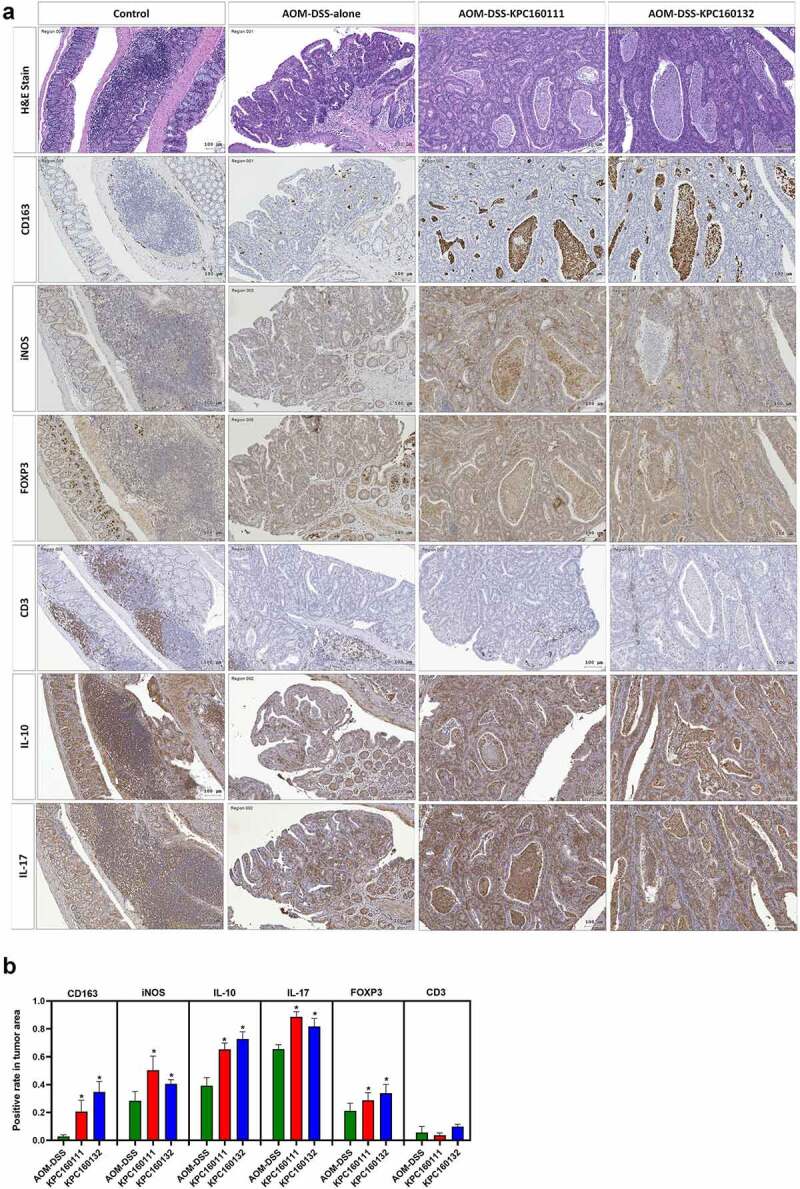
**Intratumoral distribution of immune cells in AOM-DSS mice with and without ST11 *K. pneumoniae* co-administration**. (a) Consecutive sections of Swiss colon rolls were subjected to H&E stain and were immunostained with specific antibodies against CD163, iNOS, Foxp3, CD3, IL-10, and IL-17. Representative images from the control or the AOM-DSS mice, which were treated alone (middle-left panel) or co-administrated with KPC160111 (middle-right panel) or KPC160132 (right panel), are presented. (b) The rates of cells with positive signals for each of the antibodies were quantified by HistoQuest Software at a high-powered field in 5 randomly selected areas in each of the tumors or corresponding control regions. Data are presented as mean ± SD (n = 30 in each group). *P* values were determined by two‐tailed Student’s *t*-test between AOM‐DSS-alone and AOM-DSS-KPC160111 or AOM-DSS-KPC160132. * P < .05

**Figure 5.**
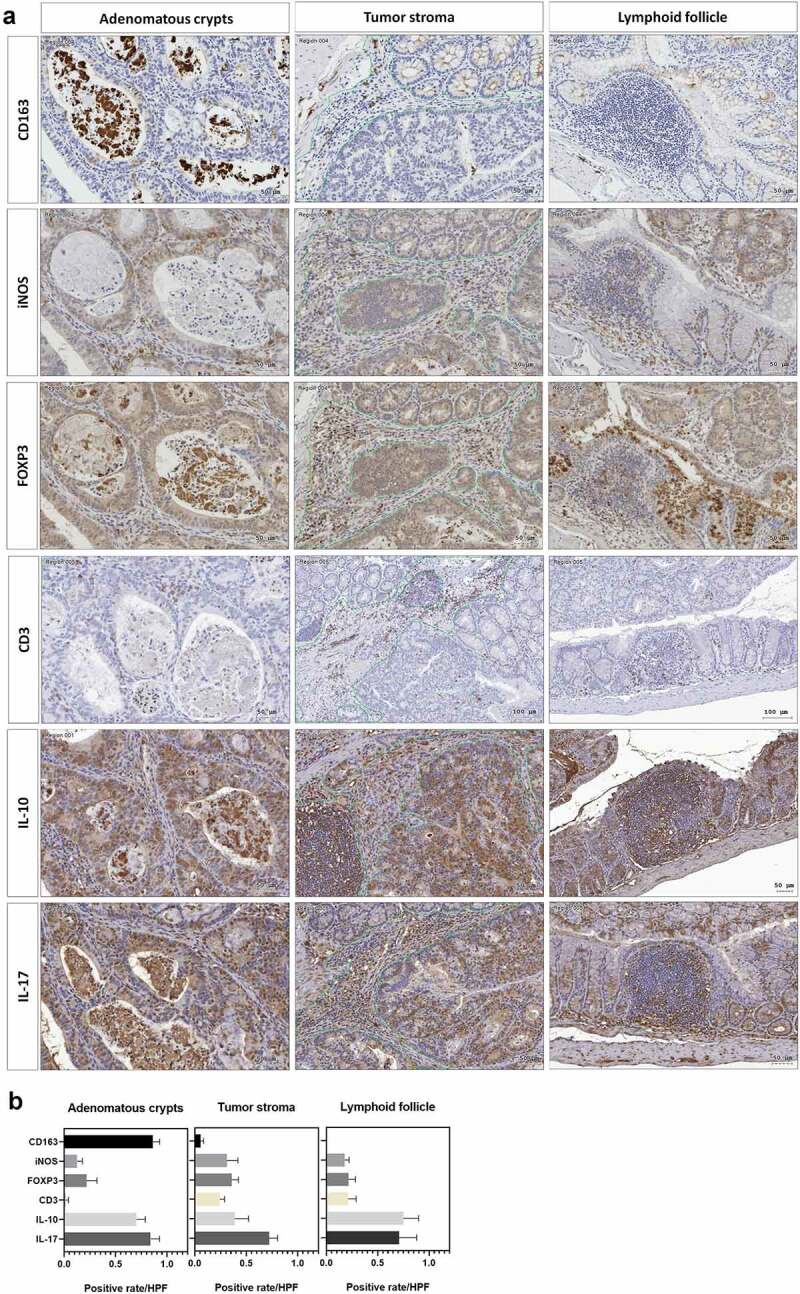
**ST11 *K. pneumoniae* promoted the accumulation of immune cells in different sub-tumoral regions**. (a) Representative images showing the distribution of immune cells in three sub-tumoral regions, adenomatous crypts, tumor stroma, and lymphoid follicles, of the adenomas developed in the AOM-DSS mice with ST11 *K. pneumoniae* co-administration. (b) HistoQuest Software quantified positive rates of CD163^+^, iNOS^+^, IL-10^+^, IL-17^+^, or Foxp3^+^ immune cells in each sub-tumoral region high-powered field in 5 randomly selected areas for ≥3 tumors of individual mice (n = 6). Data are presented as mean ± SD

Adenomas are the most common premalignant lesions which will remain stable or progress to high-degree dysplasia and finally develop into carcinomas. During tumorigenesis, the transition of host immunity from immunosurveillance to immunosuppression may facilitate malignancy. Given that *K. pneumoniae* is known to deploy immune evasion tactics during infections,^28^ we next investigated whether the deterioration of colorectal tumorigenesis by ST11 *K. pneumoniae* was associated with immunosuppressive responses. The distribution of several subsets of immune cells was inspected throughout the entire colon Swiss roll sections by immunohistochemistry with specific antibodies. Within the tumors, the abundances of cells positive for CD163, iNOS, FOXP3, IL-10, and IL-17 were significantly increased in the AOM-DSS-ST11 *K. pneumoniae* mice (Figure 4a,b). The various transitions of macrophages, including epithelioid cells, giant cells, and foam cells, which accumulated in the lumen of ST11 *K. pneumoniae*-associated adenomatous crypts, were mostly CD163^+^ (Figure 3d-f). Some CD163^+^ macrophages exhibited PAS positivity (Figure 3g). The production of IL-10 by the CD163^+^ macrophages (Figure 5a,b) suggested that ST11 *K. pneumoniae* promoted the accumulation of M2-like tumor-associated macrophages in the lumens of adenomatous crypts. Also, ST11 *K. pneumoniae* increased the densities of the tumor-infiltrating FoxP3^+^ cells, which distributed primarily in the stromal region of adenomas (Figure 5a,b). Besides the clustering of immune cells positive for IL-10 or IL-17 in lymphoid follicles, the IL-10 and IL-17-expressing epithelial cells were also detected within the adenomas of the AOM-DSS-ST11 *K. pneumoniae* mice (Figure 5a,b).

### *ST11* K. pneumoniae *promoted the accumulation of CD163^+^ macrophages in the peritoneal cavity*

**Figure 6.**
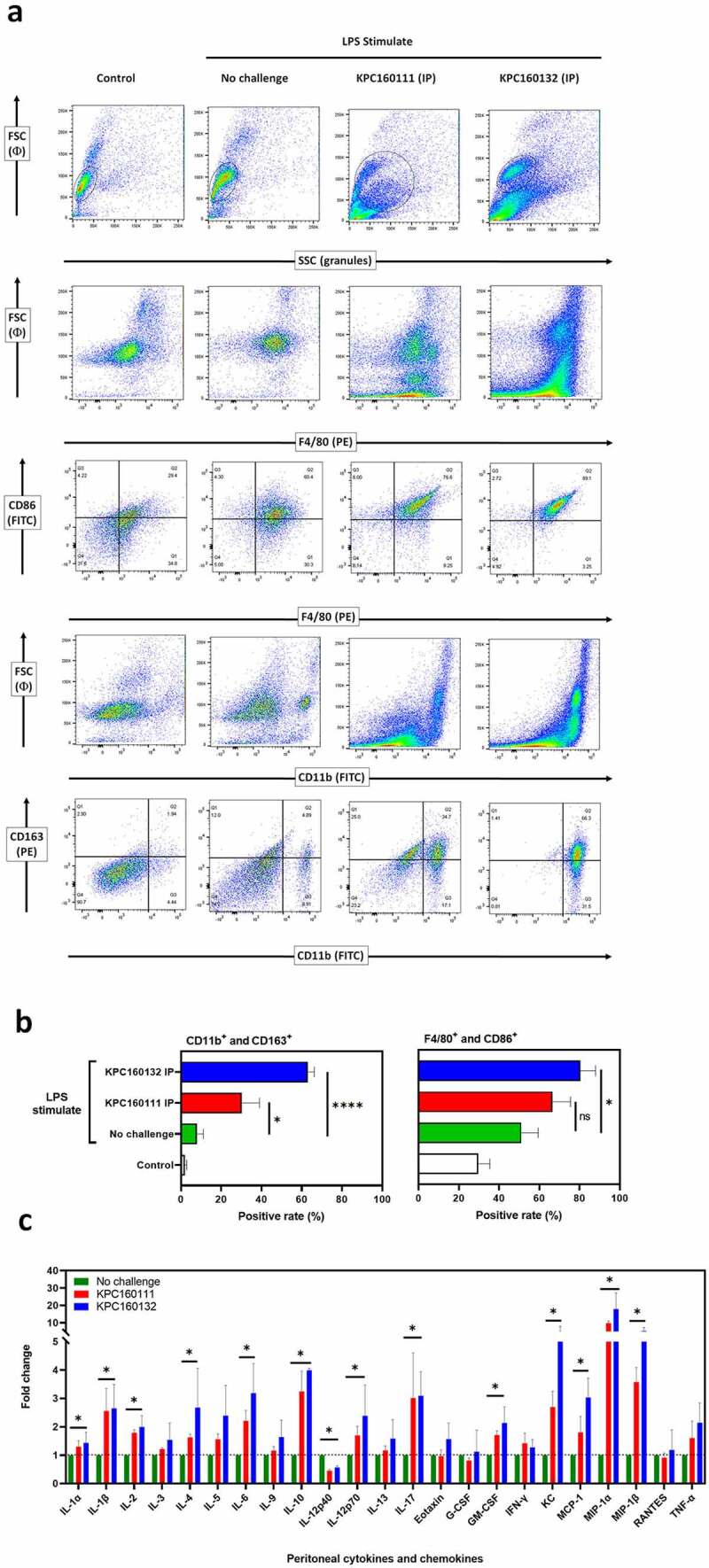
**ST11 *K. pneumoniae* polarized peritoneal macrophages toward a pro-tumoral M2-like phenotype**. (a) Flow cytometry analysis of peritoneal cells, harvested from LPS-stimulated BALB/c mice with and without the post-challenge of 1 × 10^9^ CFU of KPC160111 or KPC160132. Rat anti-mouse fluorescent antibodies, including PE-F4/80, FITC-CD86, FITC-CD11b, and PE-eFluor 610-CD163, were used. Flow cytometry data were analyzed with FlowJO *v*10.7. (b) The percentages of CD11b^+^CD163^+^ and F4/80^+^ CD86^+^ peritoneal cells are presented as mean ± SD (n = 6/group). *P* values were determined by two‐tailed Student’s *t*-test between LPS-stimulated alone (green bar) and post-challenge with KPC160111 (red bar) or KPC160132 (blue bar). ns: no significance; * P < .05; **** *P* < .0001. (c) Fold changes of the peritoneal levels of cytokines and chemokines in response to the intraperitoneal challenge of KPC160111 (red bar) or KPC160132 (blue bar) in LPS-stimulated mice (green bar)

Tumor-associated macrophages (TAMs) are defined as macrophages populated in the tumor microenvironment, which favor tumor development by suppressing anti-tumor immunity. In progressive tumors, TAMs share many characteristics with alternatively activated macrophages (M2). Since ST11 *K. pneumoniae* might exert its tumor-promoting effects through activating CD163^+^ TAMs, we examined whether ST11 *K. pneumoniae* skewed M2 polarization of peritoneal macrophages. Non-lethal *E. coli* O55:B5 LPS was intraperitoneally (IP) injected into groups of 8-wk old BALB/c male mice to increase peritoneal macrophages. After 16 hours, the LPS-stimulated mice were intraperitoneally (IP) challenged with KPC1601111 or KPC160132 (1 × 10^9^ CFU). At 1.5 hours post-IP, peritoneal cells were isolated and analyzed for the expression of various surface markers. In the LPS-stimulated mice, the percentage of CD163^+^ myeloid cells (CD11b^+^) was significantly increased from 8.1 ± 1.8% to 30.0 ± 5.1% and 63.0 ± 1.8% by the IP challenge of KPC160111 and KPC160132, respectively (Figure 6a,b). Besides, accumulation of CD86^+^ F4/80^+^ cells was also noted in the mice with ST11 *K. pneumoniae* challenge, particularly in the KPC160132 group. This result suggested that ST11 *K. pneumoniae* promoted not only the skew of M2 polarization but also the increase of M1 macrophages. The ST11 *K. pneumoniae*-induced immune responses were reflected by the changes in peritoneal levels of cytokines and chemokines. As shown in Figure 6c, the levels of macrophage-recruiting chemokines, including monocyte chemoattractant protein-1 (MCP-1/CCL2), macrophage inflammatory protein 1-alpha (MIP-1α/CCL3), and macrophage inflammatory protein-1β (MIP-1β/CCL4), were significantly elevated by the intraperitoneal challenge of ST11 *K. pneumoniae*. The increase in cytokines associated with M2 macrophages (IL-4, IL-10, and IL-17) was more significant than that of M1 macrophages (IL-1, INF-γ, and TNF-α).

### *ST11* K. pneumoniae *skewed M2 polarization of RAW264.7 cells through the activation of the STAT6-KLF4-IL10 pathway*

**Figure 7.**
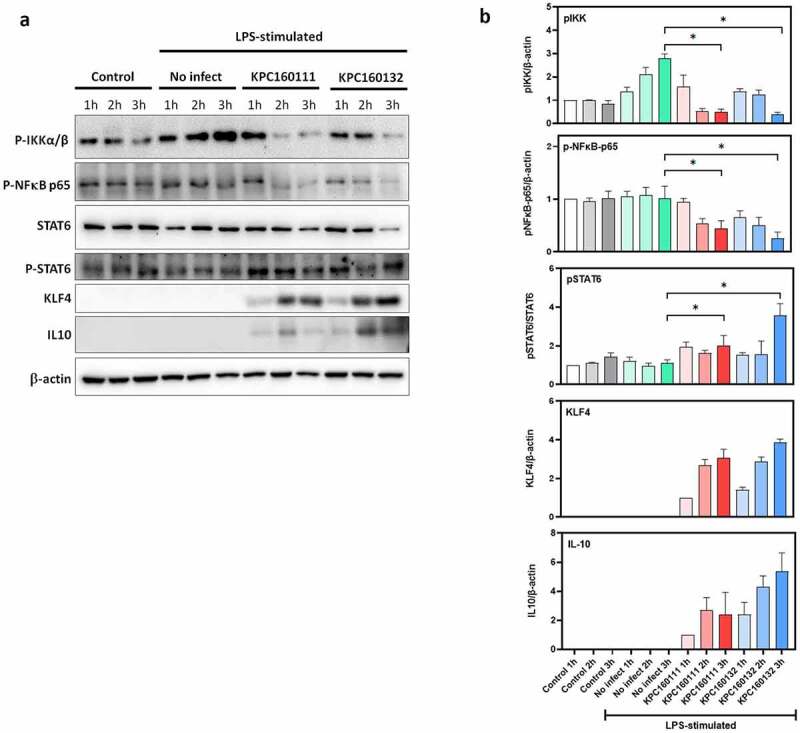
**ST11 *K. pneumoniae* polarized RAW264.7 macrophages toward M2-like signaling**. (a) LPS-stimulated RAW264.7 cells were left uninfected or infected with KPC160111 or KPC160132 at an MOI of 100 for 1, 2, and 3 hours. Total cell lysates (50 μg) were used for the Western blotting analysis of phospho-IKKαβ-S176/180, phospho-NF-κB p65, STAT6, phospho-STAT6-Tyr641, KLF4, and IL-10. (b) Relative levels of individual proteins were estimated by measuring band densities with Image J software and normalized with that of β-actin, except phospho-STAT6. Data are shown as fold changes ± SD vs. control group, except KLF-4 and IL-10, shown as fold changes vs. KPC160111-1 h. *P* values were analyzed with two-tailed Student’s *t*-test between LPS-stimulated alone (green bar) and infection with KPC160111 (red bar) or KPC160132 (blue bar). * P < .05

RAW264.7 is a macrophage-like cell line derived from BALB/c mice, which has been used as an appropriate macrophage model. Confluent RAW264.7 cells were stimulated by LPS overnight and then infected with KPC160111 or KPC160132 at a multiplicity of infection (MOI) of 100 for 1–3 hours. The protein levels of phosphor-IKKαβ, phosphor-NF-κB p65, STAT6, phosphor-STAT6, KLF4, and IL10 were examined by Western blotting with specific antibodies. The levels of LPS-stimulated phosphorylation of IKKαβ and NF-κB p65 were decreased by ST11 *K. pneumoniae*, which reached a statistical significance at 3 h post-infection (Figure 7a,b). On the other hand, ST11 *K. pneumoniae* activated the phosphorylation of STAT6 and induced the expression of Krüppel-like factor 4 (KLF4), which upregulated IL-10 expression. This result suggested that ST11 *K. pneumoniae* polarized RAW264.7 macrophages toward an M2 phenotype via the inhibition of IKK-dependent NF-κB pathway and the upregulation of IL10 expression through the activation of STAT6 and KLF4.

### *ST11* K. pneumoniae *exacerbated colitis-associated gut dysbiosis*

**Figure 8.**
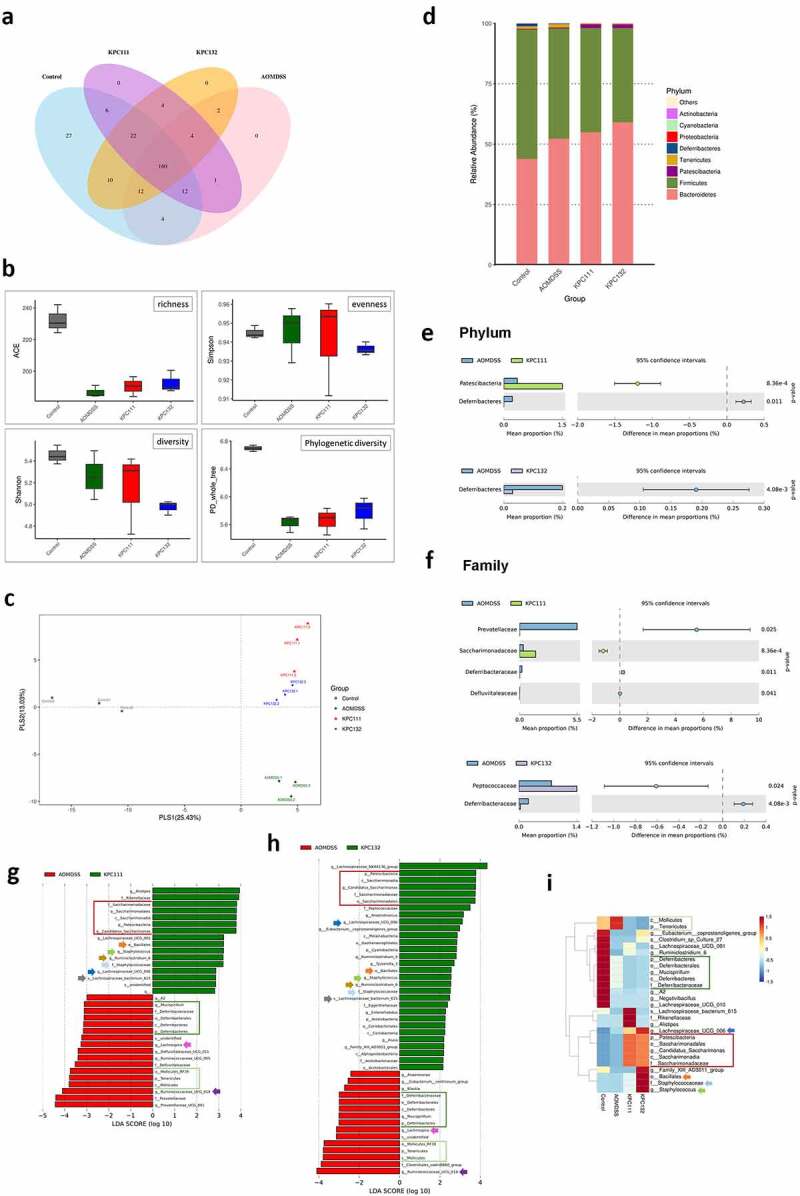
**ST11 *K. pneumoniae* exacerbated the colitis-associated gut dysbiosis**. Fecal pellets were collected from individual mice of the control (n = 3), AOM‐DSS-alone (n = 3), AOM-DSS-KPC160111 (n = 3), and AOM-DSS-KPC160132 (n = 3) groups at week 17 and subjected to the 16S microbial sequencing analysis. (a) The number of shared operational taxonomic units (OTUs) between groups is shown in the Venn diagram. (b) The α-diversity between groups is expressed by ACE richness, Simpson’s evenness, Shannon’s diversity, and phylogenetic diversity (PD_whole_tree) index. (c) The β-diversity on unweighted Unifrac distances are presented by partial least-square discriminant analysis (PLS-DA), showing the structure of bacterial communities between individual mice, which were untreated (gray-colored), or treated with AOM-DSS-alone (green-colored), AOM-DSS-KPC160111 (red-colored), or AOM-DSS-KPC160132 (blue-colored). (d) Relative abundance of OTUs per group is expressed by the bar chart showing percentages at the phylum level. The OTUs showing significant differences in relative abundance (%) in microbiomes of AOM-DSS-alone (blue bar) or with KPC160111 (green bar) or KPC160132 (purple bar) co-administration is shown at the phylum level (e) and the family level (f) with 95% confidence intervals and *P* values. The OTUs that were differently enriched were determined using LDA effect size (LEfSe) analysis. Compared to the AOM-DSS-alone (red bar), the differentially enriched OTUs with LDA score ≥2.0 by KPC160111 or KPC160132 administration (green bar) are shown in (g) and (h), respectively. The biomarker OTUs with LDA ≥2.0 for each of the four groups are highlighted in the heatmap (i)

Dysbiosis of the gut microbiome is linked to the development of colorectal cancer. The promotion of colorectal tumorigenesis by ST11 *K. pneumoniae* might also be attributed to alterations in gut microbial communities. We further investigated changes in fecal microbiomes for three representative mice in each group at the end of the experiment (Figure 1a) by 16S microbial sequencing analysis. Based on the 97% identity threshold, observed operational taxonomic units (OTUs) for the normal control, AOM-DSS-alone, AOM-DSS-KPC160111, and AOM-DSS-KPC160132 groups were 354 ± 2, 270 ± 14, 258 ± 11, and 273 ± 14, respectively. A total of 160 OTUs were shared among all groups, as expressed by the Venn diagram (Figure 8a). The α-diversity indices were calculated and compared between groups. Regardless of whether ST11 *K. pneumoniae* was co-administered, the AOM-DSS treatment reduced the richness and phylogenetic diversity of gut microbiomes (Figure 8b). The phylogenetic distances between groups were examined to assess whether the microbiome composition was significantly affected. The AOM-DSS-alone microbiomes from individual mice were clustered and separated from those with ST11 *K. pneumoniae* co-administration (red spots: KPC160111; blue spots: KPC160132) along the vertical axis PLS2 of the least-squares discriminant analysis (PLS-DA) (Figure 8c). The relative abundance of the top 10 OTUs per group is expressed by the bar chart showing percentages at the phylum level (Figure 8d). Both ST11 *K. pneumoniae* strains significantly caused the depletion of the *Deferribacteres* phylum (Figure 8e) and the *Defferribacteraceae* family (Figure 8f) (*P* < .05, Student’s *t*-test). To further analyze if any OTUs could be considered bacterial biomarkers associated with ST11 *K. pneumoniae*, we performed the linear discriminant analysis (LDA) effect size (LEfSe) analyses.^29^ With the cutoff value of the absolute LDA score (log_10_) >2.0, 10 OTUs were significantly altered in the AOM-DSS-alone group (Supplementary Figure S2a, b). Under the AOM-DSS setting, KPC160111 and KPC160132 enriched 16 and 30 OTUs (Figure 8g,h), respectively, which are highlighted on the phylogenetic tree (cladogram) (Supplementary Figure S2 c, d) and the heatmap (Figure 8i). The ST11-associated biomarker OTUs, which were commonly identified in the KPC160111 and KPC160132 groups, included the *Candidatus Saccharimonas* lineage (from genus to the *Patescibacteria* superphylum), the *Staphylococcus* lineage (from genus to the *Bacillales* order), *Ruminiclostridium_6, Lachnospiraceae_UCG_006*, and *Lachnospiraceae_bacterium_615*.

## Discussion

ST11 dominated CRKP isolates in Asia. Besides serving as a trafficker of multi-drug resistance, ST11 *K. pneumoniae* also possessed an ability to promote colorectal tumorigenesis. As we demonstrated in this study, two representative ST11 strains, KPC160111 (KL47_ST11) and KPC160132 (KL64_ST11), polarized macrophages toward an M2-like phenotype through the activation of the STAT6-KLF4-IL10 axis. Accumulation of M2-like macrophages inside adenomatous crypts created an immunosuppressive microenvironment favoring the growth of colorectal tumors induced in the AOM-DSS mouse model. *K. pneumoniae* can silently colonize both healthy individuals and ill patients.^30^ Under normal physiologic conditions, GI colonization with ST11 *K. pneumoniae* (KPC160111 and KPC160132) did not predispose BALB/c mice to the development of CRC (Figure 1e). However, after the induction of colitis-associated tumorigenesis by AOM-DSS treatment, the growth of colorectal adenomas was significantly promoted by ST11 *K. pneumoniae* (Figure 1f-h). This result suggested that ST11 *K. pneumoniae* was not a carcinogen *per se* but could act as a promoter for the growth of tumors that are already present. The AOM-DSS-induced condition extended the period of ST11 *K. pneumoniae* colonization (Figure 1c,d). The positive effect of inflammation on *K. pneumoniae* colonization has also been demonstrated previously for KpIA565, a nonpathogenic strain GI-enriched in mice with DSS-induced colitis.^31^

Inflammation drives gut dysbiosis, compromising colonization resistance against *K. pneumoniae*. The AOM-DSS treatment altered the composition of the GI microbiome in male BALB/c mice (Supplementary Figure S2a, b). Several of the AOM-DSS-associated biomarker OTUs were also demonstrated in other studies, including *Bacteroides*,^32^ *Muribaculaceae*,^33^ *Defluviitaleaceae-UCG011*, and *Christensenellaceae_R7-group*.^34^ Under the inflammatory circumstances, ST11 *K. pneumoniae* found a niche to colonize and induced further changes of the microbiome. The *Candidatus Saccharimonas* lineage (from genus to phylum) and the *Staphylococcus* lineage (from genus to order) were significantly enriched by ST11 *K. pneumoniae* (Figure 8g-i and Supplementary Figure S2c, d). As formerly named Candidate division TM7, *Candidatus Saccharimonas* belongs to the newly defined superphylum *Patescibacteria*. TM7 prevails in natural habitats^35^ and the human oral cavity.^36^ Several human diseases, such as periodontitis and inflammatory bowel disease, have been demonstrated to correlate with TM7 enrichment positively (31). *Staphylococcus* spp. are frequent commensals of the nares and skin and are considered transient colonizers in healthy individuals’ oral and GI tract. Increased abundance of *Staphylococcus* was detected in the oral and gut microbiota in patients with periodontitis^37^ and celiac disease,^38^ respectively. On the other hand, ST11 *K. pneumoniae* reduced the population size of the *Mucispirillum* lineage (from genus to phylum) and the *Mollicutes* lineage (from order to phylum) (Figure 8g-i). *Mollicutes* correlated with increased energy harvest efficiency in lean individuals^39^ and had relatively lower abundances in the gut microbiomes of CRC patients.^40^ *Mucispirillum schaedleri* is a core member of *Mucispirillum* colonizing the mucus layer of intestines,^41^ which has been demonstrated for protecting C57BL/6 J mice against *Salmonella*-induced colitis.^42^ In our model, the AOM-DSS-driven reduction of *Mucispirillum spp*. was deteriorated by ST11 *K. pneumoniae* colonization. Given that CRKP could invade the mucus layer,^43^ ST11 *K. pneumoniae* could gain a foothold in the colon mucosa by outcompeting the resident *Mucispirillum* spp.

CRC development largely attributes to environmental factors, including the complex interaction between GI microbiota and host immunity.^44^ The most significant feature that linked the colorectal tumors to ST11 *K. pneumoniae* was the formation of adenomatous crypts (Figures 3 and 5). The crypt-inside cells were primarily CD163^+^ macrophages. Through the production of IL-10, the CD163^+^ macrophages induced immune suppression in the surroundings of adenomatous crypts, which favored tumor growth and progression to high-grade dysplasia.^45^ *K. pneumoniae* is known as a master tactician in immune evasion through the induction of high-level IL-10.^46^ The requirement of IL-10 for *K. pneumoniae* infections has been previously demonstrated that the administration of anti-IL-10 antibodies improved the survival of mice with *K. pneumoniae*-triggered pneumonia.^47^ Inversely, the overexpression of IL-10 accelerated the mortality of infected mice.^48^ IL-10 has a two-way immunomodulatory effect. In addition to anti-inflammation, IL-10 can also repress immune effector cells to create an immunosuppressive microenvironment. With the loss of anti-inflammatory control, mice deficient in IL-10 overproduce Th1 cytokines,^49^ develop spontaneous colitis,^50^ and are prone to produce intestinal tumors in the *Apc^Min^* genetic background.^51^ In *Apc^Min/+^; Il10*^−/−^ mice, *K. pneumoniae* 51–5 increased colonic tumors with significant infiltration of CD3^+^ cells in lamina propria.^20^ Involvement of T cell responses, which was derepressed by the deletion of IL-10, was suggested in the *K. pneumoniae* 51-5-promoted colon tumorigenesis. Although the *Apc^Min/+^; Il10*^−/−^ mice have been widely used in the colitis-associated CRC study, it is difficult for us to use this model to investigate the two-way roles of IL-10. In wild-type BALB/c mice, the presence of IL-10 inhibited inflammation and Th1 responses. Therefore, we did not observe an increase in the number of CD3^+^ cells in response to ST11 *K. pneumoniae* (Figures 4 and 5), even though this model allowed us to investigate the two-way roles of IL-10 in the CRC progression.

*K. pneumoniae* is a stealth pathogen exploiting the anti-inflammatory properties of IL-10 to attenuate host immunity. Nevertheless, our knowledge regarding how it interacts with immune cells to stimulate the production of IL-10 is still restricted. IL-10 is a pleiotropic cytokine produced by myeloid and lymphoid cells.^52^ In an *ex vivo* porcine lung perfusion model, *K. pneumoniae* Kp52145 skewed alveolar macrophages toward an M2-like state with CD163 expression and IL-10 production.^53^ In line with this, we found that both KPC160132 (KL64) and KPC160111 (KL47) elicited the enrichment of CD11b^+^ CD163^+^ macrophages in the peritoneal cavity, and the peritoneal level of IL-10 was simultaneously elevated (Figure 6a-c). The signaling molecules triggered by ST11 *K. pneumoniae* were further investigated in the RAW264.7 macrophages (Figure 7a,b). Upon exposure to KPC160111 or KPC160132, the level of LPS-induced phosphorylation of the IκB kinase (IKK) α/β was reduced, and therefore the activation of the main M1 transcription factor, NF-κB, was inhibited. In contrast, the phosphorylation of STAT6 and the expression of KLF4 were simultaneously enhanced by ST11 *K. pneumoniae*. STAT6 is the key transcriptional activator for M2 polarization,^54^ and KLF4 cooperates with STAT6 to induce the M2 genetic program.^55^ The increased production of IL-10, regulated by KLF4,^56^ acted in an autocrine manner to reinforce the M2 phenotype of RAW264.7 macrophages. Collectively, these results suggested that ST11 *K. pneumoniae* polarized macrophages toward a pro-tumoral M2-like phenotype through the inhibition of IKK-NFκB and the activation of STAT6-KLF4-IL-10 axis.

**Figure 9.**
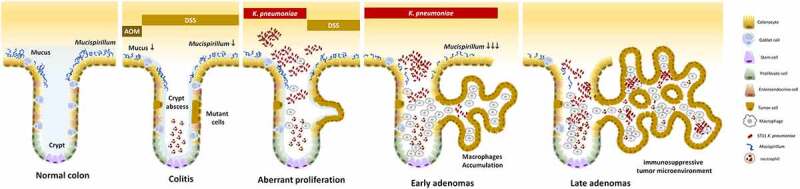
**Schematic representation of a hypothesized process of ST11 *K. pneumoniae*-promoted tumorigenesis in an AOM-DSS-induced colitis model**. Accumulation of the AOM-induced DNA mutations caused the transition of a small portion of colonocytes into the tumorigenic state. Recurrent episodes of DSS-generated inflammation reduced the production of mucus and the abundance of mucus-associated *Mucispirillum*, and elicited crypt abscesses (neutrophils accumulation in the lumen of crypt). Under this circumstances, ST11 *K. pneumoniae* expansion promoted the accumulation of CD163^+^ macrophages inside the crypt lumen. Through the massive production of IL-10 by these macrophages, the surroundings of the crypts became immunosuppressive areas, which help the evasion of ST11 *K. pneumoniae* from the host immunity. On the other hand, it allowed the aberrant growth of tumor cells, contributing to the early-to-late transition of colorectal adenomas

Unlike the crypts under the inflammatory condition which are filled with neutrophils,^57^ the ST11 *K. pneumoniae*-triggered adenomatous crypts were filled the aggregates of macrophages, which formed a structure similar to a granuloma. In response to ST11 *K. pneumoniae*, the crypt-inside macrophages underwent a series of morphological changes, including epithelioid cell differentiation, fusion into multinucleated giant cells, and transformation to foam cells (Figure 3d,e). Unlike the granulomas of Crohn’s disease and intestinal tuberculosis, which are mainly composed of inflammatory M1 macrophages,^58^ anti-inflammatory M2-like macrophages constituted the core of ST11-*K. pneumoniae*-associated granulomas (Figures 3 and 5). The production of IL-10 by accumulating M2-like macrophages contributed to developing an immunosuppressive microenvironment favoring tumor growth. Moreover, cells in the adenomatous epithelium also increasingly produced IL-10 and IL-17, which could simultaneously act on the crypt-inside macrophages to reinforce the pro-tumoral M2 phenotype.^59^ The schematic representation of a hypothesized process of ST11 *K. pneumoniae*-promoted tumorigenesis is shown in Figure 9.

*K. pneumoniae* colonizes the GI tract; however, it is not considered an enteropathogen. Most people who carry *K. pneumoniae* experience no symptoms of colitis. Instead of launching a lethal attack to destroy the gut mucosa, ST11 *K. pneumoniae* creates an immunosuppressive niche favoring its extended stay in the GI tract. The polarization of M2 macrophages by ST11 *K. pneumoniae* is harmless in the normal colon, but if it occurs in the tissues that have already become cancerous, it subverts the anti-tumor immunity toward a tolerogenic milieu favoring tumor growth and progression. Given that *K. pneumoniae* Kp52145, a ST66 strain, could activate the M1-to-M2 shift of macrophages,^53^ we considered that neither the M2-skewing ability nor the tumor-promoting effect was restricted to ST11 lineage. Further studies are needed to gain a comprehensive understanding of the pro-carcinogenic potential of different lineages of *K. pneumoniae* and the mechanisms through which they promote colorectal tumorigenesis.

## Materials and methods

### Bacterial strains

ST11 *K. pneumoniae*, KPC160111 and KPC160132, were isolated in our previous studies.^26,27,60^ The genome of these two strains was fully sequenced and deposited in GenBank with the accession numbers CP029689.1 and CP040023.1, respectively. KPC160111, which was isolated from an 81-yr female patient who suffered from urinary tract infection in 2014, belonged to ST11_KL47 and carried six plasmids, including two distinct *bla*_KPC-2_ and *bla*_OXA-48_ plasmids. KPC160132, isolated from the fecal carriage of a long-term hospitalized patient in 2015, was classified ST11_KL64 with the carriage of *bla*_OXA-48_ on its chromosome. Mid-log phase cultures of *K. pneumoniae* KPC160111 or KPC160132 were maintained in Luria-Bertani (LB) broth, with a determined density of CFU/ml were used for subsequent experiments. The *in vitro* growth of these two ST11 strains was unaffected by 0.2–5% DSS, which was the drug added in drinking water in the mouse experiments (Supplementary Figure S3a-c).

### The AOM-DSS mouse model

Male BALB/c mice were purchased from BioLasco (Taiwan Co., Ltd) at the age of 7-wk-old, allowed to acclimatize in the animal house for one week, and randomized into six groups. Mice in groups 1 through 3 were given an intraperitoneal injection of 5 mg of azoxymethane (AOM; Wako, Japan) kg body weight on days 1, 4, 8, and 11. Four days after the 4^th^ injection of AOM, the mice were subjected to 3 rounds of dextran sodium sulfate (DSS) (MW: 36000–50000, MP Biomedicals, USA) treatment. Each DSS round consisted of administering 2% (w/v) DSS in drinking water for 7 days, followed by a 14-day recovery period with regular water. Mice in groups 4 through 6 were untreated with AOM-DSS, serving as the age-matched normal control. At weeks 5, 8, and 11, mice in groups 2, 5, and 3, 6, were orally inoculated with 1 × 10^9^ CFU of mid-log-phase KPC160111 and KPC160132, respectively. Before each challenge of *K. pneumoniae*, mice drank water supplemented with streptomycin (2 mg/ml) for 3 consecutive days. Other mice that were not challenged with *K. pneumoniae*, including the control and AOM-DSS-alone groups, also drank streptomycin-water for three consecutive days at the corresponding time points. Fecal pellets were collected from individual mice to determine *K. pneumoniae* CFU on imipenem (10 μg/ml)-M9 minimal media plates at indicative time points. At week 17, all animals were sacrificed by isoflurane overdose. For each of the mice, the large bowel, from ileocecal junction to the anal verge, was excised and flushed with 5% glycerol in sterile saline, measured length, and longitudinally cut open. The number and size of colonic polyps were examined under a stereomicroscope by two investigators. The size of the polyp was calculated as length × width, which was measured with a micro caliper. Based on the size, polyps were categorized into three classes, sporadic (<4 mm^2^), small (4 ~ 9 mm^2^), and large (≥9 mm^2^).

### Colon histopathology and immunochemistry analysis

Colons were prepared as a Swiss roll, fixed in 10% of formalin, and processed for paraffin embedding. The hematoxylin and eosin (H&E) sections were examined by experienced pathologists based on the criteria described.^61^ For immunohistochemistry analysis, consecutive sections were de-waxed with xylene, rehydrated in graded alcohol, antigen-retrieval with 0.01 M of citric buffer (pH6.0), and hybridized with specific antibodies against Ki-67 (Abcam #ab15880, rabbit polyclonal, dilution 1:150), β-catenin (CST #8480, rabbit monoclonal, dilution 1:100), carbonic anhydrase II (Abcam #ab124687, rabbit monoclonal, dilution 1:100), CD163 (Abcam #ab182422, rabbit monoclonal, dilution 1:100), iNOS (Abcam #ab15323, rabbit polyclonal, dilution 1:150), Foxp3 (BioLegend clone poly6238, rabbit monoclonal, dilution 1:100), CD3 (Abcam #ab16669, rabbit monoclonal, dilution 1:100), IL-10 (ABclonal #A2171, rabbit polyclonal, dilution 1:150), and IL-17 (Abcam #ab79056, rabbit polyclonal, dilution 1:150). The images of whole Swiss roll colon sections were scanned and acquired using the TissueFAXS plus system (TissueGnostics GmbH). Positive rates of the cells showing positive signals against a specific antibody in the indicated regions were quantified with the HistoQuest Software provided by TissueGnostics GmbH at a high-powered field in 5 randomly selected areas in each of the tumors or corresponding control regions.

### Flow cytometry analysis of peritoneal cells

Groups of 8-wk-old male BALB/c mice were intraperitoneally injected with 200 ul PBS containing with and without LPS (1 mg/Kg of weight; *E. coli* O55:B5; Sigma). After 16 hours, the LPS-stimulated mice were intraperitoneally challenged with 1 × 10^9^ CFU of *K. pneumoniae* KPC160111 or KPC160132. Peritoneal cells were harvested by injecting 5 mL of PBS at 1.5 h post-bacterial challenge, and the supernatants were collected for cytokine/chemokine analysis. After RBC lysis and washing, peritoneal cells were treated with Mouse Seroblock FcR (Bio-Rad Laboratories # BUF041) for 20 min at 4°C to block the Fc receptors and then incubated with rat anti-mouse fluorescent antibodies to comparatively analyze the distribution of the myeloid cells in response to *K. pneumoniae* challenge. The antibodies used were PE-conjugated anti-F4/80 (eBioscience #12-4801-82), FITC-conjugated anti-CD11b (eBioscience #11-0112-82), FITC-conjugated anti-CD86 (eBioscience #11-0862-82), and PE-eFluor 610-conjugated anti-CD163 (eBioscience #61-1631-82). After incubation at 4°C for 1 h, cells were washed and analyzed with a BD FACS Canto II cytometer (Becton Dickinson). Flow cytometry data were analyzed with FlowJO *v*10.7 (Becton Dickinson).

### Cytokine/chemokine analysis of peritoneal fluids

The levels of interleukin (IL)-1α, IL-1β, IL-2, IL-3, IL-4, IL-5, IL-6, IL-9, IL-10, IL-12 (p40), IL-12 (p70), IL-13, IL-17, eotaxin, G-CSF, GM-CSF, IFN-γ, KC, MCP-1, MIP-1α, MIP-1β, RANTES, and TNF-α in the peritoneal fluids, collected from the control mice and the LPS-stimulated mice, which were post-challenged with and without KPC160111 or KPC160132, were quantified using the Bio-Plex Pro Mouse Cytokine 23-Plex Immunoassay (Bio-Rad; Model: M60009RDPD) according to the instruction manual.

### Western blotting analysis

RAW 264.7 murine macrophage (BCRC #60001, Taiwan) cells were maintained in 5% CO_2_ at 37°C in Dulbecco’s Modified Eagle’s Medium with 10% fetal bovine serum. After seeding to 6-well culture plates at a density of 2 × 10^6^ cells/well, RAW264.7 cells were stimulated with 1 μg/ml LPS (*E. coli* O55:B5; Sigma #L2880) for 16 hours. The LPS-stimulated RAW.264.7 cells were harvested after transient infections with KPC160111 or KPC160132 at an MOI of 100 for 1, 2, and 3 hours. At least 10^7^ cells were collected and lysed with 1 ml of lysis buffer (PBS, 1% Triton X-100, protease, and phosphatase inhibitors). Protein concentrations of the total lysates were measured with Pierce™ BCA Protein Assay Kit (Thermo Scientific™ #23225). Fifty micrograms of total proteins were separated over 10–15% SDS-PAGE gels and transferred onto Immun-Blot® PVDF Membranes (Bio-Rad #1620177). After blocking with 5% nonfat dry milk (or 3% BSA for phospho-antibodies) in TBST, the blots were washed and hybridized at 4°C overnight with specific antibodies at 1:1000 dilution, including STAT6 (Cell Signaling Technology #5397), phospho-STAT6-Tyr641 (Cell Signaling Technology #56554), phospho-IKKαβ-S176/180 (ABclonal # AP0546), phospho-NF-κB p65 (Cell Signaling Technology #3033), KLF4 (ABclonal # A6640), IL-10 (ABclonal # A2171), and β-actin (Novus # NB600-501). Relative levels of individual proteins were estimated by measuring band densities with Image J software and normalized with that of β-actin, except phospho-STAT6. Data are shown as fold changes ± SD vs. control group, except KLF-4 and IL-10, shown as fold changes vs. KPC160111-1 h. Two-tailed *P* values were analyzed with Student’s *t*-test between LPS-stimulated alone and infection with ST11 K. pneumoniae. * P < .05

### 16S rRNA amplicon sequencing and analysis of fecal microbiome

Total genomic DNA was extracted from fecal samples collected from individual mice retrieved at the end of the experiment, using QIAamp DNA Stool Mini Kit (QIAGEN) following the manufacturer’s protocol. The V3-V4 hypervariable region of 16S rDNA was amplified using the bacterial-specific forward (5ʹ TCG TCG GCA GCG TCA GAT GTG TAT AAG AGA CAG CCT ACG GGN GGC WGC AG 3ʹ) and reverse (5ʹ GTC TCG TGG GCT CGG AGA TGT GTA TAA GAG ACA GGA CTA CHV GGG TAT CTA ATC C 3ʹ) primer set using the 16S Metagenomic Sequencing Library Preparation Kit (Illumina). Index adapters were added to the amplicons with the Nextera XT Index Kit, and the constructed libraries were subjected to 2 × 300 bp paired-end sequencing runs on the Illumina MiSeq platform. Amplification and sequencing services of the 16S rRNA gene were provided by Welgene Biotech Co., Ltd. (Taiwan). Clean reads were obtained after raw FASTQ reads filtered with Bowtie 2^62^ and the removal of PhiX control by Trimmomatic.^63^ Qualified sequences (effective tags) were clustered into de novo operational taxonomic units (OTUs) by >97% similarity using USEARCH.^64^ The OTU sequences were annotated using the SILVA reference database (SSURef NR 99 release 123).^65^ Sequences that could not be assigned to a specific taxonomy level were labeled as unclassified. Unweighted Pair-group Method with Arithmetic Means (UPGMA) Clustering was performed as a hierarchical clustering method to interpret the distance matrix using average linkage and was conducted by QIIME.^66^ The α-diversity indices, including Chao, Ace, Shannon, and observed OTUs, were calculated using the MOTHUR. The β-diversity on weighted and unweighted Unifrac distances were calculated by QIIME.^67^ Cluster analysis on the linear model, principal component analysis (PCA), and the nonlinear model, NMDS, was performed using the FactoMineR and ggplot2 packages in R software. Partial least-square discriminant analysis (PLS-DA), implemented in MetaboAnalyst, was also performed.^68^ The linear discriminant analysis (LDA) effect size (LEfSe) analyses were conducted by LEfSe software, with the cutoff value as the absolute LDA score (log_10_) ≥2.0.^29^ Metastats were calculated by R software. Statistical significance between groups was examined using permutational multivariate analysis of variance based on the observed Unifrac distance matrix relative to 10,000 randomly rearranged distance matrices.^69^

### Statistical analysis

Statistical analyses were performed using GraphPad Prism 9.0 (GraphPad Software Inc.). Comparison between two groups indicated was conducted by Student’s *t*-test. A significant difference between groups was considered when two-tailed *P* < .05.

### Ethics statement

All animal experiments were conducted following the Guide for the Care and Use of Laboratory Animals of the National Research Council Committee.^70^ The protocols of animal experiments were approved by the Institutional Animal Care and Use Committee of Chung Shan Medical University (permit no. 2172 and 2315).

## Supplementary Material

Supplemental Material

## Data Availability

Metagenome sequence information is available at NCBI under the Sequence Read Archive (SRA) database with accession no PRJNA728507. All data generated or analyzed during this study are included in this article. Any additional information will be made available from the corresponding author on reasonable request.
