# Supplementary material for: Two ST11 Klebsiella pneumoniae strains exacerbate colorectal tumorigenesis in a colitis-associated mouse model
Source: Gut Microbes. 2021 Oct 4;13(1):1980348. doi: 10.1080/19490976.2021.1980348 (PMC8496539; doi:10.1080/19490976.2021.1980348)
Supplement: Supplemental Material [file KGMI_A_1980348_SM5261.pdf]

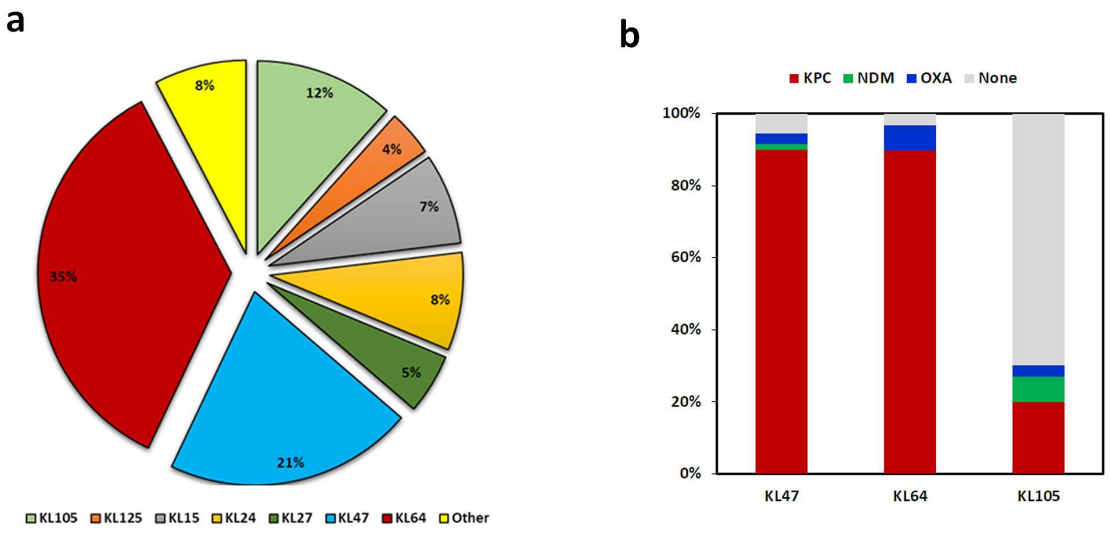

Supplementary Figure 1. KL64 and KL47 constituted the majority of ST11 CRKP. (a) A total of 857 ST11 *K. pneumoniae* genomes publicly available in October 2019 were included. Among the 28 different types of the capsule (K)-loci identified in ST11 *K. pneumoniae*, KL64 and KL47 predominated over others, occupying 35% and 21%, respectively. **(b)** The percentage of genomes with the presence of carbapenemase genes, including bla_{KPC} (red-color), $bla_{OXA\text{-}48}$ (blue-color), and bla_{NDM} (green-color), was calculated in the ST11 sublineages expressing KL47, KL64, or KL105. If a bla_{KPC}-positive genome simultaneously had other carbapenemase genes, it was calculated as bla_{KPC}-positive.

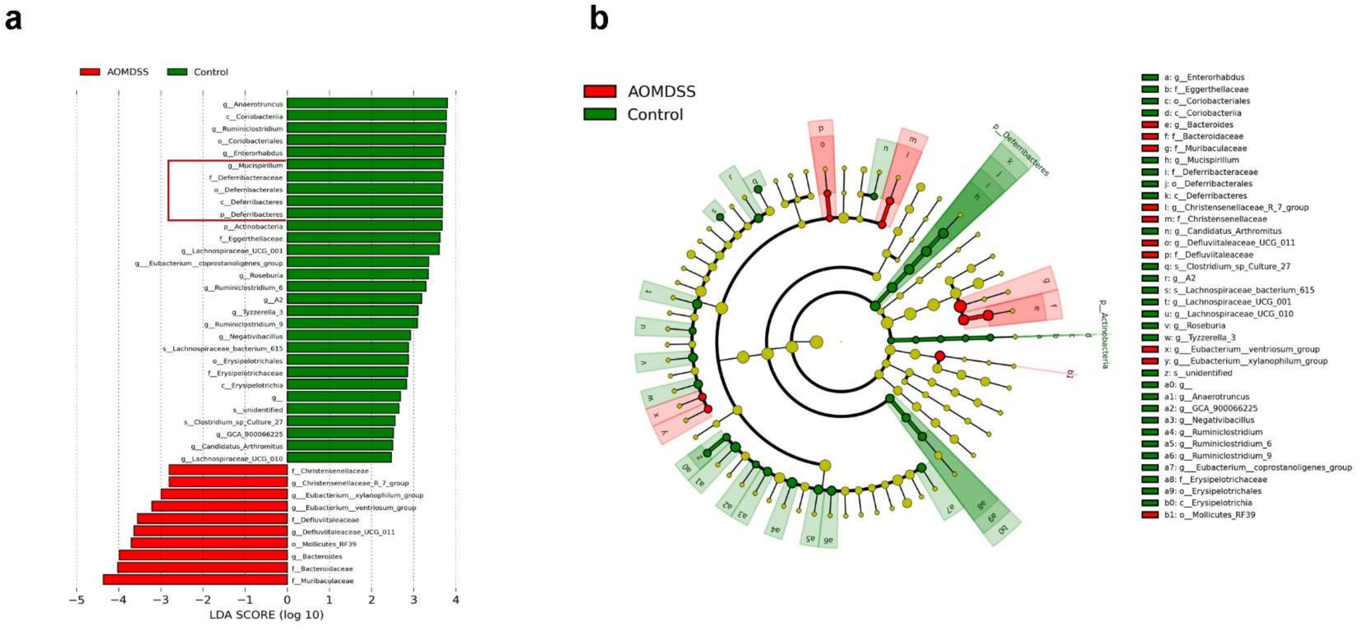

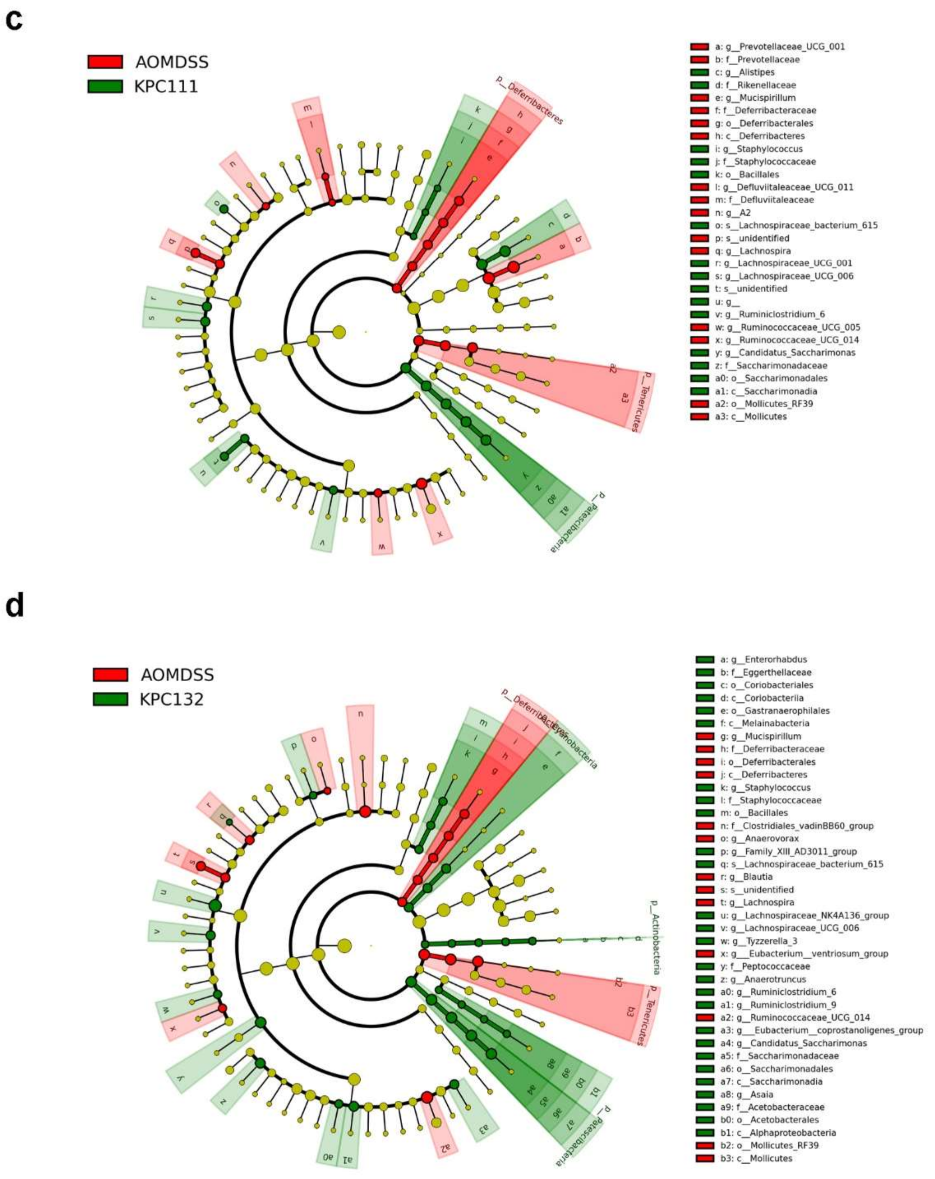

Supplementary Figure 2. Taxa which were differently enriched in AOM-DSS-alone (red-color) or the control mice (green color) were determined using LDA effect size (LEfSe) analysis. The differentially enriched taxa with LDA score (≥ 2.0) are shown in **(a)** and highlighted in the phylogenetic tree **(b)**. Compared to the AOM-DSS-alone group, taxa which were enriched by KPC160111 or KPC160132 with LDA score ≥ 2.0 are highlighted in the phylogenetic tree, **(c)** and **(d)**, respectively. In the phylogenetic tree (cladogram), the size of nodes correlates to the taxa abundance.

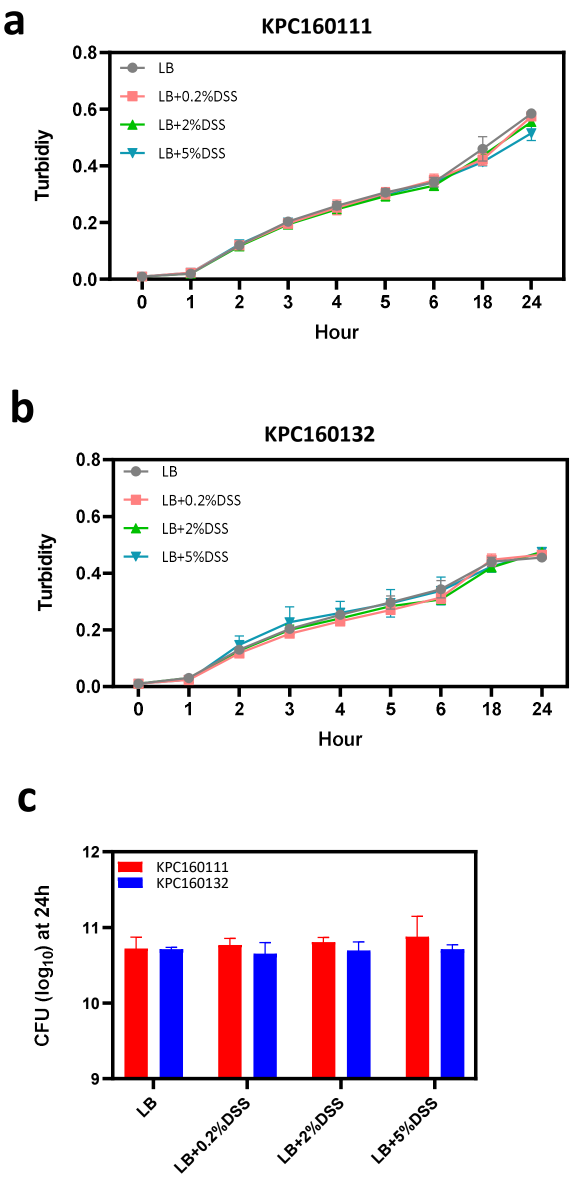

Supplementary Figure 3. *In vitro* growth of ST11 *K. pneumoniae* in the presence of dextran sodium sulfate (DSS). Forty microliters of overnight LB cultures of KPC160111 and KPC160132 were inoculated into 2 ml LB with and without 0.2%, 2%, or 5% DSS at 37°C with shaking (200 rpm). Turbidity of bacterial culture was measured at 1, 2, 3, 4, 5, 6, 18, and 24 h using Microscan Turbidity Meter (SIEMENS), and the values of mean ± SD are presented in (a) KPC160111 and (b) KPC160132. (c) The colonies forming units (CFUs) were determined after a 24-h growth with the standard plate count method and the average log_{10}CFU values are shown.
